# Microstructure and Mechanical Properties of Particulate Reinforced NbMoCrTiAl High Entropy Based Composite

**DOI:** 10.3390/e20070517

**Published:** 2018-07-10

**Authors:** Tianchen Li, Bin Liu, Yong Liu, Wenmin Guo, Ao Fu, Liangsheng Li, Nie Yan, Qihong Fang

**Affiliations:** 1 State Key Laboratory for Powder Metallurgy, Central South University, Changsha 410083, China; 2 College of Mechanical and Energy Engineering, Shaoyang University, Shaoyang 422000, China; 3 YuanMeng Precision Technology (Shenzhen) Institute, Shenzhen 518000, China; 4 College of Mechanical and Vehicle Engineering, Hunan University, Changsha 410082, China

**Keywords:** high entropy alloys, metal matrix composites, mechanical properties

## Abstract

A novel metal matrix composite based on the NbMoCrTiAl high entropy alloy (HEA) was designed by the in-situ formation method. The microstructure, phase evolution, and compression mechanical properties at room temperature of the composite are investigated in detail. The results confirmed that the composite was primarily composed of body-centered cubic solid solution with a small amount of titanium carbides and alumina. With the presence of approximately 7.0 vol. % Al_2_O_3_ and 32.2 vol. % TiC reinforced particles, the compressive fracture strength of the composite (1542 MPa) was increased by approximately 50% compared with that of the as-cast NbMoCrTiAl HEA. In consideration of the superior oxidation resistance, the P/M NbMoCrTiAl high entropy alloy composite could be considered as a promising high temperature structural material.

## 1. Introduction

Recently the concept of high-entropy alloys (HEAs), consisting of four or more principle metallic elements in equiatomic or near-equiatomic ratios, has attracted considerable interest owing to its tendency to form solid solution with outstanding mechanical and functional properties [1,2,3,4,5,6]. By introducing refractory elements including group IV (Ti, Zr, and Hf), V (V, Nb, and Ta), and VI (Cr, Mo, and W) in HEAs, the developed refractory HEAs (RHEAs) possess high melting temperature, outstanding strength and hardness, high thermal stability, and softening resistance at elevated temperatures, which opens up the possibilities of the alloy developments satisfying structural demands at high temperature.

Since the first WMoTaNb RHEA was reported in 2010, many literatures have been focused on the preparation methods and mechanical properties of RHEAs [7,8]. For example, the BCC WMoTaNb and WMoTaNbV HEAs maintained yield strength of 405 and 477 MPa at 1600 °C respectively, which were much higher than that of Inconel 718 and Haynes 230 [9]. However, most of these RHEAs are generally characterized with high density and poor high temperature oxidation resistance. Such drawbacks are the current bottleneck for utilizing RHEAs as structural materials. In order to improve the oxidation resistance properties of the RHEAs, it was reported that the Ti, Al, Cr, and Si elements are essential for the formation of protective oxide scales. Compared with the traditional alloys, it was widely reported that the RHEAs possessed low diffusivity as well as a wider range of composition design [10]. In addition, it was also reported that the density of RHEAs could be significantly reduced by the additions of light elements, such as Ti [11,12]. Therefore, on the basis of design principles above, a new RHEA with equiatomic composition of NbMoCrTiAl was designed by B. Gorr et al. [13,14]. The as-cast alloy with low density (7.58 g/cm^3^) exhibits excellent oxidation resistance. The declining oxidation rate at 1100 °C was mainly due to the formation of Al, Cr-rich oxide layers. However, the compressive strength of the as-cast NbMoCrTiAl alloy was reported to be as low as 1010 MPa and its high temperature strength is insufficient.

As reported in the literature, most of the RHEAs were prepared by the arc melting process. These as-cast alloys generally exhibited a dendritic structure with severe composition segregation [11,12,13]. Powder metallurgy (P/M), which is considered a high efficiency technique, is widely used to synthesize non-equilibrium materials. The materials characterized with fine grains are without chemical segregation and evaporation of alloying elements caused by arc-melting [15,16]. As reported, P/M WMoTaNbV shows fine grains of micrometer-scale and ultra-high compressive yield strength of 2612 MPa [17]. Therefore, it is an effective way to prepare high performance RHEAs.

Another prospective candidate for the high strength structural materials was the metal matrix composite (MMCs). The high performance was mainly attributed to the interaction between the particles and dislocations, called dispersion strengthening effect and second-phase strengthening effect. As the NbMoCrTiAl matrix is selected, the crucial challenge is to select the suitable strengthening phase and numbers of papers can be used as references [18,19,20,21]. The reinforcement particles include carbides (TiC, SiC), oxides (Al_2_O_3_, Y_2_O_3_), nitrides, (Si_3_N_4_, BN), and borides (TiB_2_, LaB_6_). There are mainly two ways to prepare carbides and oxides strengthened composites. One simple measure is to add them directly, another way is to form in-situ carbides or oxides by introducing C and O elements, which can effectively reduce material defects like pores and cracks. It was reported that the TiC and Al_2_O_3_ are also commonly used as strengthening phases because of their intrinsic high strength characteristics [22,23].

In this work, in order to further increase the strength of NbMoCrTiAl RHEA, the P/M composite with dispersed carbides and oxides was developed through ball milling and spark plasma sintering (SPS) method. The phase constitutions, microstructures, and mechanical properties of the NbMoCrTiAl HEA composite were investigated in detail.

## 2. Materials and Methods

Powders of Nb, Mo, Cr, Ti, and Al with purity higher than 99.5 wt. % and particle size of ≤45 μm were used as starting materials. The elemental powder was mixed in equiatomic composition and then processed by high energy planetary ball milling for 39 h at 300 rpm in a pure argon atmosphere (YXQM-2L, MITR, Changsha, China). Tungsten carbide vials and balls were utilized as the milling media with a ball-to-powder ratio of 10:1. The empirical addition amount of stearic acid was 3.5 wt. %. The stearic acid was introduced as process control agent (PCA) to prevent cold welding as well as particle agglomeration, and also acted as additive for generating particulate reinforced HEA composite. Subsequently, the as-milled powders were consolidated by SPS (D25/3, FCT, Munich, Germany) under 30 MPa axial pressure at 1700 °C with the heating rate of 100 K/min. During the sintering process, the sample was held at the sintering temperature for 30 min. Finally the sample cooled to room temperature in furnace.

The samples after sintering were analyzed by X-ray diffraction (XRD, D/MAX-2250, Rigaku, Tokyo, Japan) with a Cu Kα radiation at 40 kV and 200 mA. The microstructure of the composite was characterized by field emission scanning electron microscope (FESEM, Quanta FEG250, FEI, Hillsboro, OR, USA). The approximate volume fraction and average grain size of different phases was evaluated by at least ten images using Image Pro software. The phase composition and element distribution were investigated with electron probe microanalysis (EPMA, EPMA-1600, JEOL, Tokyo, Japan). Microhardness of the sample was measured by a Vickers microhardness tester (MicroMet-5104, Buehler, Lake Bluff, IL, USA) with an indenter load of 100 g and the holding time was 15 s. Cylindrical specimens of compression tests (4 mm in diameter and 6 mm in height) were cut and machined from the sintered bulk. Room temperature compression tests were carried out on an Instron-3369 (INSTRON, Norwood, MA, USA) universal testing machines at strain rate of 10^−3^ s^−1^.

## 3. Results

### 3.1. Microstructure and Phase Evolution after SPS

**Figure 1:**
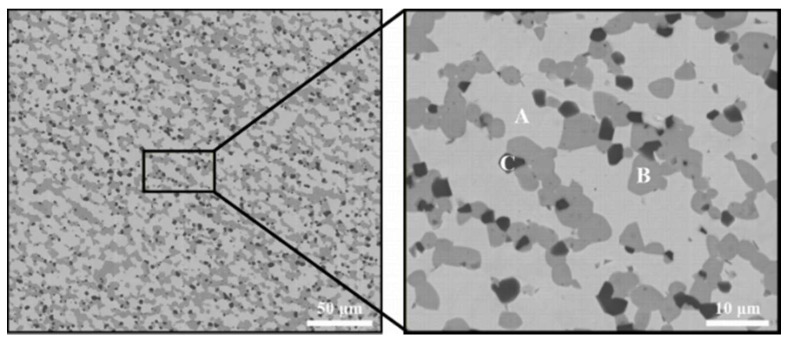
Microstructure of the NbMoCrTiAl composite after spark plasma sintering at 1700 °C for 30 min under a pressure of 30 MPa.

The general microstructure of the HEA composite is shown in Figure 1. The specimens are highly densified by the reaction sintering process without visible pores. The composite presented three obviously identifiable contrasts, namely light, grey, and black regions (marked as A, B, and C). The volume fraction of the light phase, grey phase, and black phase are 60.8%, 32.2%, and 7.0%, respectively. Since the content of carbon and oxygen cannot be measured accurately by EDS, EPMA experiment was employed to analyze the chemical composition in these areas (Table 1). The results indicate that the region A consists of only 10.66 at. % Ti and 16.79 at. % Al, while Ti and Al atoms are enriched in the region B and C, respectively. Noticeably, the region C in Figure 1 was enriched in Al and O, indicating the formation of Al_2_O_3_.

**Figure 2:**
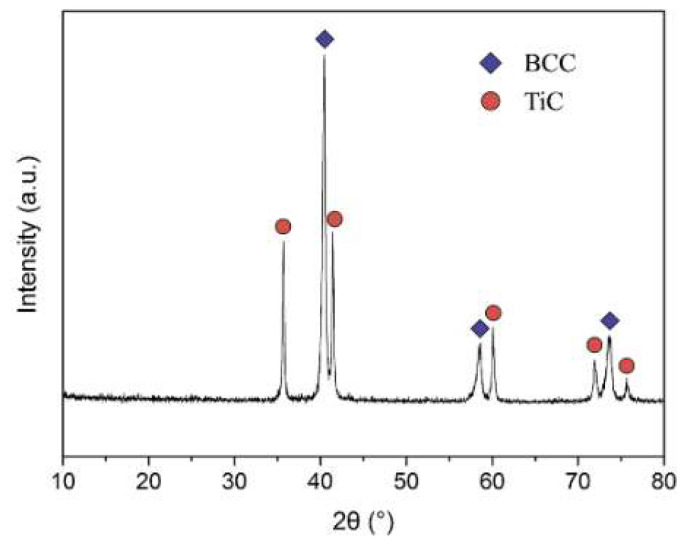
XRD pattern of the NbMoCrTiAl composite.

The XRD patterns of the RHEA composite were illustrated in Figure 2. Two types of characteristic diffraction peaks could be identified, indicating the present material is primarily composed of BCC crystalline structure (a = 0.315 nm) with diffraction peaks at 2θ = 43.66°, 50.82° 74.67° accompanied with a certain amount of TiC phase (a = 0.435 nm) as the reinforcing phase. Al_2_O_3_ cannot be detected by X-ray analysis, which was probably ascribed to the limit detection of 5%. In consideration of the EDS analysis results, it could be concluded that the light, grey, and black phase were identified as BCC HEA phase, TiC phase, and Al_2_O_3_ phase, respectively. The average particle size of TiC and Al_2_O_3_ were measured to be approximately 4.60 μm and 1.57 μm, respectively.

**Figure 3:**
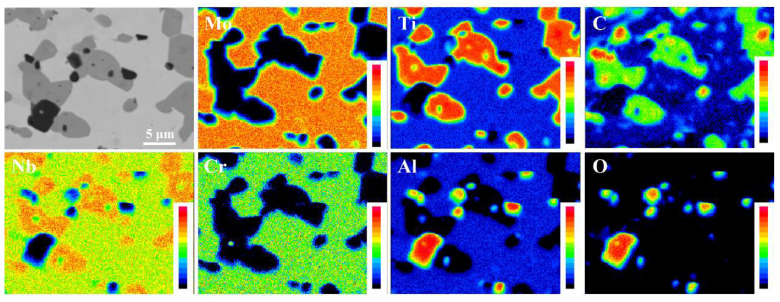
The elemental mapping of the composite obtained by electron probe microanalysis (EPMA).

In order to further investigate the element distribution, the element mapping of the RHEA composite was shown in Figure 3. It was clear that the Mo and Cr elements are enriched almost entirely in the light region (BCC phase). On the contrary, the Ti and C elements are enriched almost entirely in the grey region (TiC phases) with certain amount of Nb, while O and Al are enriched in the black region (Al_2_O_3_ phase). No apparent debonding between dispersed particles and the HEA matrix could be found, which suggested that the secondary phase grains and the HEA grains possessed good bonding.

### 3.2. Mechanical Properties

**Figure 4:**
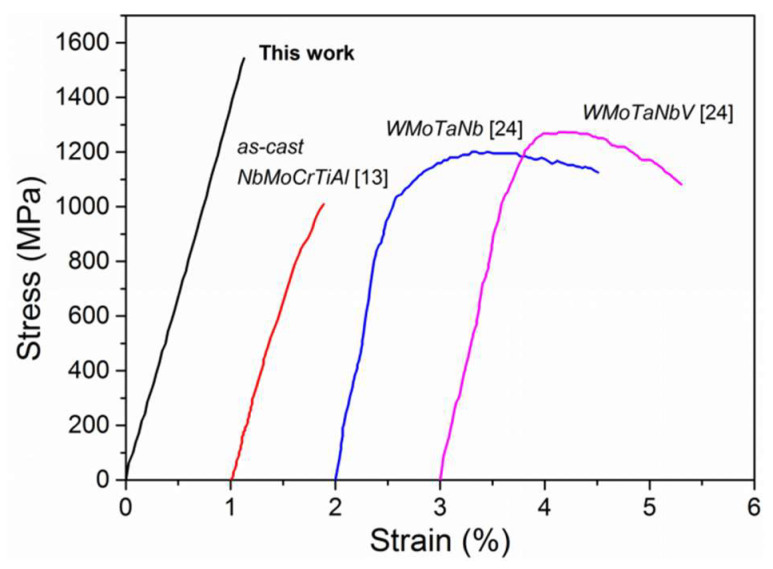
The room temperature engineering stress-strain curves of the composite and typical RHEAs reported in the literatures [13,24].

The room temperature engineering stress-strain curves of the Nb_20_Mo_20_Cr_20_Ti_20_Al_20_ composite was given in Figure 4. Despite no ductility, the fracture strength of the composite was 1542 MPa, increasing by more than a half than that of the as-cast alloy (1010 MPa) [13]. The fracture strength was also much higher than that of the traditional WMoTaNb and WMoTaNbV RHEAs [24], which have yield strengths of 1246 MPa and 1058 MPa, respectively, as shown in Figure 4. As a result of the plasticity and oxidation resistance of the NbMoCrTiAl matrix at high temperature, it was concluded that the P/M NbMoCrTiAl HEA composite had relatively superior mechanical properties and could be considered as a potential high temperature structural material [25].

**Figure 5:**
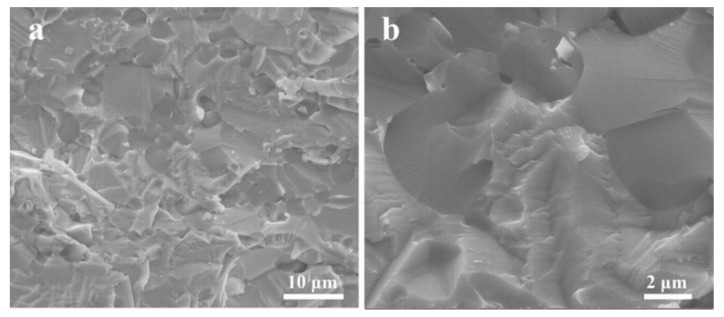
Microstructure features of the fracture surface of the composite deformed at room temperature (**a**) at low magnifications and (**b**) high magnifications.

Microstructure features of the fracture surface of the composite deformed at room temperature are shown in Figure 5. Cleavage fracture is featured by river pattern, which could be clearly seen in Figure 5. This means that the NbMoCrTiAl HEA composite shows typical cleavage fracture mode without ductility. The analysis result is in accordance with the deformation curves above.

## 4. Discussion

### 4.1. Phase Formation

Although a lack of HEA phase diagrams limit the availability of thermodynamic data, some thermodynamic properties, such as atomic size, melting point, mixing entropy, mixing enthalpy, and valence electron concentration, can be used as criteria to predict the phase formation in the HEA system. According to the empirical rules, BCC solid solutions were generally formed in HEA systems under the following certain conditions. (1) The electron concentration (VEC) was lower than 6.87 [26]; (2) a new parameter combining effects of entropy and enthalpy (Ω) was proposed to be more than 1 and atomic size differences (δ) was less than 6.6% [27]. δ, Ω, and VEC are calculated to be 5.4, 2.92 and 4.80 respectively, so BCC solid solution could be anticipated, which tallied well with the research results produced by H. Chen et al. [13]. Guo et al. [28] further proposed that all ductile alloys have a VEC ≤ 4.4, while all brittle alloys have a VEC ≥ 4.6. Therefore, the NbMoCrTiAl RHEA should be without ductility and it is indeed a type of brittle RHEA.

As mentioned above, BCC phase, TiC phase, and Al_2_O_3_ phase can be observed in Figure 1. The actual composition of BCC phase is Nb_20.3_Mo_31.0_Cr_19.3_Ti_10.7_Al_16.7_, ignoring the contributions made by interstitial elements of carbon and oxygen. According to the actual composition of the BCC high entropy phase, all the parameters calculated for the composition presented in this paper are summarized (δ = 5.6, Ω = 3.46, VEC = 4.96). According to the data, the HEA phase should be of BCC structure, and Figure 2 verifies accuracy of the prediction. As a result, we could confirm that such non-equiatomic phase composition is able to enhance the solid solution especially at high temperature [29].

Combining high energy ball-milling of powder precursors with the following SPS compaction, the HEA composite was successfully synthesized in this work. The addition of stearic acid can be used as an effective way for the preparation of composite materials. During the process of high energy ball-milling, PCA is used to not only prevent cold-welding, but also act as the source of the elements carbon and oxygen. Table 2 shows the values of H_mix_ (kJ/mol) calculated by Miedema’s model for atomic pairs between elements with Nb, Mo, Cr, Ti, Al, and C. As shown in Figure 2, this certain concentration of Nb incorporated during grain growth agrees with the large solubility of solute Nb-carbide in solvent TiC mainly because of their largest negative value of Hmix and identical crystal structure. While Mo, Cr, and Al could not dissolve in the TiC due to their relatively weak cohesive force [30].

### 4.2. Strengthening Mechanism

The composite created a kind of material with highly improved compressive strength, increasing from 1010 MPa to 1542 MPa. This remarkable increase in mechanical properties can be attributed to the following reasons. The most important one is the effect of second phase strengthening. It can be found that introduction of brittle ceramic reinforcements would significantly increase mechanical properties of the material. As listed in Table 3, the yield strength of (FeCrNiCo)Al_0.7_Cu_0.5_ base HEA reached 630 MPa accompanied by a high strain of 42.7%. With the addition of 10 vol. % TiC, the yield strength increased to 1290 MPa, while plastic strain dropped to 29.2% [31]. FeCoCrNiMn high entropy alloy matrix nanocomposite with addition of SiC was prepared by hot isostatic pressing, which result in the value of yield strength increased to 1600 MPa [18]. In addition, the interphase boundary between the in-situ reinforced particles and the solid solution matrix is free of cracks and pores, resulting in the more possibilities for the design of advanced high strength structural components.

Another main factor is grain refinement strengthening mechanism. The grain size of the as-homogenized NbMoCrTiAl phase is about 100 μm, while that of the composite is much lower. This is because the PM process is an effective approach to refine grains compared to arc-melting [16]. In addition, the hard phases evenly distributed at the grain boundary, acting as the barrier of grain growth. Praveen et al. [32] reported that the grain growth of the FeCoCrNi composite could be obviously suppressed by two-phase mixture (FCC-HEA and carbide) microstructure.

The third reason is the influence of solution strengthening. It is true that the solution element could have remarkable strengthening effect. In this study, the BCC phase contains 0.69 at. % O and 1.28 at. % C, which may cause significant increase in strength because these two interstitial atoms have a substantially smaller size than any of the metal atoms in the HEA. These interstitial atoms will produce a substantial strain field, with which gliding dislocations will interact. There have been studies on the influence of interstitial elements, such as element C, O, and N. Wang et al. [33,34] reported that the addition of 1.1 at. % carbon to a novel single-phase FCC Fe_40.4_Ni_11.3_Mn_34.8_Al_7.5_Cr_6_ HEA not only markedly increased the yield strength from 159 MPa to 355 MPa, but also led to a 25 % increase in the elongation to fracture. An increase in yield strength was also observed in the C-dissolved FeCoCrNi fabricated by selective laser melting [35]. With regard to the carbide, the lattice constant of TiC (0.435 nm) is slightly larger than pure TiC (0.433 nm). The different crystal parameter may result from the solution of atoms with large radius, such as element Nb (r = 0.148 nm) in this composite. Therefore, we may reasonably conclude that solution-strengthening effect resulted in the improved mechanical properties.

## 5. Conclusions

1. A novel metal matrix composite based on the NbMoCrTiAl high entropy alloy (HEA) was designed by the in-situ formation method. Strengthening phases include TiC particles and Al_2_O_3_ particles. The volume fraction of the BCC phase, TiC phase, and Al_2_O_3_ phase were 60.8%, 32.2%, and 7.0%, and the average particle size of TiC and Al_2_O_3_ were measured to be approximately 4.60 μm and 1.57 μm, respectively.
2. The Mo and Cr elements are enriched almost entirely in the BCC phase. By contrast, the Ti and C elements are enriched almost entirely in the TiC phases with certain amount of Nb, while O and Al are enriched in the Al_2_O_3_ phase. No apparent debonding between dispersed particles and the HEA matrix could be found, which suggested that the secondary phase grains and the HEA grains possessed good bonding.
3. The composite created a kind of material with highly improved compressive strength (1542 MPa) compared with that of the as-cast NbMoCrTiAl alloy (1010 MPa). On the basis of microstructure features of the fracture surface, typical cleavage fracture mode was found in the composite. The remarkable increase in mechanical properties can be attributed to second phase strengthening effect, grain refinement and solid solution effect.

## Figures and Tables

**Table 1:** Chemical composition of high entropy alloy (HEA) composite determined by electron probe microanalysis (EPMA) analysis.

Region	Nb	Mo	Cr	Ti	Al	O	C
A	20.29	30.95	19.34	10.66	16.79	0.69	1.28
B	15.40	-	-	43.13	-	12.11	29.36
C	-	-	-	-	38.13	61.87	-

**Table 2:** Mixing enthalpies (kJ/mol) of unlike atomic pair [30].

	Nb	Mo	Cr	Ti	Al	C
Nb	-					
Mo	−6	-				
Cr	−7	0	-			
Ti	2	−4	−7	-		
Al	−18	−5	−10	−30	-	
C	−102	−67	−61	−109	−36	-

**Table 3:** Room temperature mechanical properties of HEAs and their composites.

Sample	σ_y_ (MPa)	σ_max_ (MPa)	ε_p_ (%)	Hardness (HV)
(FeCrNiCo)Al_0.7_Cu_0.5_ [31]	630	2270	42.7	313
(FeCrNiCo)Al_0.7_Cu_0.5_ + 10 vol. % TiC [31]	1290	2444	29.2	580
FeCoCrNiMn [18]	1180	2660	34.5	418
FeCoCrNiMn + 5 wt. % SiC [18]	1600	1930	6.8	545

## References

[1] Liu Y., Wang J., Fang Q., Liu B., Wu Y., Chen S. (2016). Preparation of superfine-grained high entropy alloy by spark plasma sintering gas atomized powder. Intermetallics.

[2] Liu B., Wang J., Liu Y., Fang Q., Wu Y., Chen S., Liu C.T. (2016). Microstructure and mechanical properties of equimolar FeCoCrNi high entropy alloy prepared via powder extrusion. Intermetallics.

[3] Zhang W., Liaw P.K., Zhang Y. (2018). Science and technology in high-entropy alloys. Sci. China Mater..

[4] Zhang Z., Axinte E., Ge W., Shang C., Wang Y. (2016). Microstructure, mechanical properties and corrosion resistance of CuZrY/Al, Ti, Hf series high-entropy alloys. Mater. Des..

[5] Xu J., Axinte E., Zhao Z., Wang Y. (2016). Effect of C and Ce addition on the microstructure and magnetic property of the mechanically alloyed fesibalni high entropy alloys. J. Magn. Magn. Mater..

[6] Shang C., Axinte E., Sun J., Li X., Li P., Du J., Qiao P., Wang Y. (2017). CoCrFeNi(W_1−x_Mo_x_) high-entropy alloy coatings with excellent mechanical properties and corrosion resistance prepared by mechanical alloying and hot pressing sintering. Mater. Des..

[7] Chen R., Qin G., Zheng H., Wang L., Su Y., Chiu Y., Ding H., Guo J., Fu H. (2018). Composition design of high entropy alloys using the valence electron concentration to balance strength and ductility. Acta Mater..

[8] Senkov O., Isheim D., Seidman D., Pilchak A. (2016). Development of a refractory high entropy superalloy. Entropy.

[9] Senkov O.N., Wilks G.B., Miracle D.B., Chuang C.P., Liaw P.K. (2010). Refractory high-entropy alloys. Intermetallics.

[10] Miracle D.B., Senkov O.N. (2017). A critical review of high entropy alloys and related concepts. Acta Mater..

[11] Liu C.M., Wang H.M., Zhang S.Q., Tang H.B., Zhang A.L. (2014). Microstructure and oxidation behavior of new refractory high entropy alloys. J. Alloys Compd..

[12] Gorr B., Azim M., Christ H.J., Mueller T., Schliephake D., Heilmaier M. (2015). Phase equilibria, microstructure, and high temperature oxidation resistance of novel refractory high-entropy alloys. J. Alloys Compd..

[13] Chen H., Kauffmann A., Gorr B., Schliephake D., Seemüller C., Wagner J.N., Christ H.J., Heilmaier M. (2016). Microstructure and mechanical properties at elevated temperatures of a new al-containing refractory high-entropy alloy Nb-Mo-Cr-Ti-Al. J. Alloys Compd..

[14] Gorr B., Mueller F., Christ H.J., Mueller T., Chen H., Kauffmann A., Heilmaier M. (2016). High temperature oxidation behavior of an equimolar refractory metal-based alloy 20Nb20Mo20Cr20Ti20Al with and without Si addition. J. Alloys Compd..

[15] Senkov O.N., Senkova S.V., Woodward C. (2014). Effect of aluminum on the microstructure and properties of two refractory high-entropy alloys. Acta Mater..

[16] Omori M. (2000). Sintering, consolidation, reaction and crystal growth by the spark plasma system (SPS). Mater. Sci. Eng. A.

[17] Kang B., Lee J., Ryu H.J., Hong S.H. (2017). Ultra-high strength WNbMoTaV high-entropy alloys with fine grain structure fabricated by powder metallurgical process. Mater. Sci. Eng. A.

[18] Rogal Ł., Kalita D., Tarasek A., Bobrowski P., Czerwinski F. (2017). Effect of SiC nano-particles on microstructure and mechanical properties of the CoCrFeMnNi high entropy alloy. J. Alloys Compd..

[19] Guo N.N., Wang L., Luo L.S., Li X.Z., Chen R.R., Su Y.Q., Guo J.J., Fu H.Z. (2016). Microstructure and mechanical properties of in-situ MC-carbide particulates-reinforced refractory high-entropy Mo_0.5_NbHf_0.5_ZrTi matrix alloy composite. Intermetallics.

[20] Fu Z., Koc R. (2017). Ultrafine TiB_2_-TiNiFeCrCoAl high-entropy alloy composite with enhanced mechanical properties. Mater. Sci. Eng. A.

[21] Riva S., Tudball A., Mehraban S., Lavery N.P., Brown S.G.R., Yusenko K.V. (2018). A novel high-entropy alloy-based composite material. J. Alloys Compd..

[22] Rogal Ł., Kalita D., Litynska-Dobrzynska L. (2017). CoCrFeMnNi high entropy alloy matrix nanocomposite with addition of Al_2_O_3_. Intermetallics.

[23] Teber A., Schoenstein F., Têtard F., Abdellaoui M., Jouini N. (2012). Effect of SPS process sintering on the microstructure and mechanical properties of nanocrystalline TiC for tools application. Int. J. Refract. Met. Hard Mater..

[24] Senkov O.N., Wilks G.B., Scott J.M., Miracle D.B. (2011). Mechanical properties of Nb_25_Mo_25_Ta_25_W_25_ and V_20_Nb_20_Mo_20_Ta_20_W_20_ refractory high entropy alloys. Intermetallics.

[25] Waseem O.A., Lee J., Lee H.M., Ryu H.J. (2017). The effect of ti on the sintering and mechanical properties of refractory high-entropy alloy TixWTaVCr fabricated via spark plasma sintering for fusion plasma-facing materials. Mater. Chem. Phys..

[26] Guo S., Ng C., Lu J., Liu C.T. (2011). Effect of valence electron concentration on stability of fcc or bcc phase in high entropy alloys. J. Appl. Phys..

[27] Zhang Y., Zhou Y.J., Lin J.P., Chen G.L., Liaw P.K. (2008). Solid-solution phase formation rules for multi-component alloys. Adv. Eng. Mater..

[28] Sheikh S., Shafeie S., Hu Q., Ahlström J., Persson C., Veselý J., Zýka J., Klement U., Guo S. (2016). Alloy design for intrinsically ductile refractory high-entropy alloys. J. Appl. Phys..

[29] Yeh J.W. (2013). Alloy design strategies and future trends in high-entropy alloys. JOM.

[30] Takeuchi A., Inoue A. (2005). Classification of bulk metallic glasses by atomic size difference, heat of mixing and period of constituent elements and its application to characterization of the main alloying element. Mater. Trans..

[31] Fan Q.C., Li B.S., Zhang Y. (2014). The microstructure and properties of (FeCrNiCo)AlxCuy high-entropy alloys and their TiC-reinforced composites. Mater. Sci. Eng. A.

[32] Sathiyamoorthi P., Basu J., Kashyap S., Pradeep K.G., Kottada R.S. (2017). Thermal stability and grain boundary strengthening in ultrafine-grained CoCrFeNi high entropy alloy composite. Mater. Des..

[33] Wang Z., Baker I., Cai Z., Chen S., Poplawsky J.D., Guo W. (2016). The effect of interstitial carbon on the mechanical properties and dislocation substructure evolution in Fe_40.4_Ni_11.3_Mn_34.8_Al_7.5_Cr_6_ high entropy alloys. Acta Mater..

[34] Wang Z., Baker I., Guo W., Poplawsky J.D. (2017). The effect of carbon on the microstructures, mechanical properties, and deformation mechanisms of thermo-mechanically treated Fe_40.4_Ni_11.3_Mn_34.8_Al_7.5_Cr_6_ high entropy alloys. Acta Mater..

[35] Zhou R., Liu Y., Zhou C., Li S., Wu W., Song M., Liu B., Liang X., Liaw P.K. (2018). Microstructures and mechanical properties of C-containing FeCoCrNi high-entropy alloy fabricated by selective laser melting. Intermetallics.

